# Evaluation of calcification distribution by CT-based textural analysis for discrimination of immature teratoma

**DOI:** 10.1186/s13048-023-01268-1

**Published:** 2023-08-28

**Authors:** Akari Nakamori, Hideaki Tsuyoshi, Tetsuya Tsujikawa, Makoto Orisaka, Tetsuji Kurokawa, Yoshio Yoshida

**Affiliations:** 1 Department of Obstetrics and Gynecology, Faculty of Medical Sciences, University of Fukui, 23-3 Shimoaizuki, Matsuoka, Eiheiji-Cho, Yoshida-Gun, Fukui 910-1193 Japan; 2 Department of Radiology, Faculty of Medical Sciences, University of Fukui, 23-3 Shimoaizuki, Matsuoka, Eiheiji-Cho, Yoshida-Gun, Fukui 910-1193 Japan

**Keywords:** Mature teratoma, Immature teratoma, CT, Fat, Calcification, Texture analysis

## Abstract

**Background:**

Mature and immature teratomas are differentiated based on tumor markers and calcification or fat distribution. However, no study has objectively quantified the differences in calcification and fat distributions between these tumors. This study aimed to evaluate the diagnostic potential of CT-based textural analysis in differentiating between mature and immature teratomas in patients aged < 20 years.

**Materials and methods:**

Thirty-two patients with pathologically proven mature cystic (*n* = 28) and immature teratomas (*n* = 4) underwent transabdominal ultrasound and/or abdominal and pelvic CT before surgery. The diagnostic performance of CT for assessing imaging features, including subjective manual measurement and objective textural analysis of fat and calcification distributions in the tumors, was evaluated by two experienced readers. The histopathological results were used as the gold standard. The Mann–Whitney U test was used for statistical analysis.

**Results:**

We evaluated 32 patients (mean age, 14.5 years; age range, 6–19 years). The mean maximum diameter and number of calcifications of immature teratomas were significantly larger than those of mature cystic teratomas (*p* < 0.01). The mean number of fats of immature teratomas was significantly larger than that of mature cystic teratomas (*p* < 0.01); however, no significant difference in the maximum diameter of fats was observed. CT textural features for calcification distribution in the tumors showed that mature cystic teratomas had higher homogeneity and energy than immature teratomas. However, immature teratomas showed higher correlation, entropy, and dissimilarity than mature cystic teratomas among features derived from the gray-level co-occurrence matrix (GLCM) (*p* < 0.05). No significant differences were observed in the CT features of fats derived from GLCM.

**Conclusion:**

Our results demonstrate that calcification distribution on CT is a potential diagnostic biomarker to discriminate mature from immature teratomas, thus enabling optimal therapeutic selection for patients aged < 20 years.

**Supplementary Information:**

The online version contains supplementary material available at 10.1186/s13048-023-01268-1.

## Introduction

In clinical practice, ovarian tumors are occasionally encountered in patients aged < 20 years. They often present with sudden severe abdominal pain due to torsion or rupture. Therefore, early intervention, including prompt clinical diagnosis and urgent surgery, is needed. However, the need to preserve fertility renders treatment of these tumors difficult. The most frequent type of ovarian tumor in this age group is germ cell tumors, of which, > 40% are malignant. Malignant germ cell tumors account for approximately two-thirds of ovarian cancers in the first two decades of life [1].

Teratomas are the most frequent type of germ cell tumor and are of two types: mature and immature. Mature teratomas are benign tumors composed of well-differentiated derivations from at least two of three germ cells (ectoderm, mesoderm, and endoderm). Moreover, immature teratomas are malignant tumors composed of immature tissue that resembles embryonic tissue but contains immature neuroectodermal components [2]. Regarding surgical resection, mature teratoma removal with simple cystectomy preserves the ovary; however, immature teratomas require at least adnexectomy even at their early stages [3]. Therefore, preoperative differentiation between both conditions is important, especially in emergencies.

Mature and immature teratomas are preoperatively differentiated based on tumor markers or the size and presence of a prominent solid component with cystic elements on imaging modalities [4, 5]. Moreover, calcification or fat distribution in the tumors differentiates them. Immature teratomas reportedly show scattered calcifications and small fatty areas; however, mature teratomas show only a few calcifications in the cyst wall [6–8]. However, no study has objectively quantified the differences in calcification and fat distributions between these tumors for preoperative differential diagnosis.

Texture analysis is a method for quantifying and quantitatively characterizing the nature of lesions in medical images and has various approaches. In most cases, the shading of pixels in the region of interest is quantified, and statistics are obtained to determine the uniformity, directionality, contrast variation, and other values that indicate homogeneity. Texture analysis can infer pathological findings or genomic subtypes and differentiate benign and malignant tumors [9–13] using imaging modalities, such as CT, MRI, or PET. Various approaches have been performed for epithelial ovarian cancer [14]. However, no study has reported its usefulness in differentiating mature from immature teratomas.

This study aimed to determine if the quantification of calcification and fat distribution by CT-based texture analysis can differentiate mature from immature teratomas in patients aged < 20 years.

## Materials and methods

### Patients

We retrospectively reviewed the medical records of 32 patients (mean age, 14.5 years; age range, 6–19 years) with pathologically proven mature cystic (*n* = 28) and immature teratomas (*n* = 4) between January 2008 and June 2021. Patients included in this study were aged < 20 years, underwent transabdominal ultrasound and/or abdominal and pelvic CT before surgery, and underwent surgery at our institution. Patients excluded were aged > 20 years and did not undergo imaging before surgery or surgery at our institution. This was a multicenter study, as 10 patients with abdominal CT data were referred from other institutions. The study protocol was approved by the Institutional Review Board of the University of Fukui Hospital (approval number: 20210730). Patients provided written informed consent to participate in the study, and anonymous clinical data were used. Patients provided written consent for the use of residual samples and were offered an opt-out option, as disclosed on the website (https://www.u-fukui.ac.jp/cont_about/disclosure/privacy/).

### CT

Abdominal and pelvic CT examinations were performed using a 64-slice multidetector CT scanner (Discovery CT 750HD; GE Medical Systems, Milwaukee, WI, USA).

### Image interpretation

Images were analyzed using the picture archiving and communication system. A three-dimensional image was constructed using the Ziostation2 software (Ziosoft, Tokyo, Japan). A gynecologic oncologist (21 years of experience) and a board-certified radiologist (24 years of experience), each with double certifications and specializing in gynecological imaging, evaluated the CT images retrospectively and reached a consensus. Images were evaluated for the following parameters: a) maximum diameter, b) mean diameter, c) volume, d) maximum diameter of calcifications, e) number of calcifications, f) maximum diameter of fats, and g) number of fats. The threshold for calcified lesions was set at a CT density of 130 Hounsfield units [15]. Additionally, fat was characterized by a CT density of –144 to –20 Hounsfield units [16]. Both readers were blinded to the results of the other imaging studies, histopathological findings, and clinical data. Each dataset was reviewed as the consensus decision of both readers after a minimum interval of 3 weeks to avoid any decision threshold bias due to reading-order effects.

### Texture analysis and feature extraction

Patients’ CT images without contrast agent were exported from the picture archiving and communication system in DICOM format and uploaded into the LIFEx software version 7. 2. 0 (LITO, CEA, Inserm, CNRS, Univ. Paris-Sud, Université Paris Saclay) [17]. The whole process was performed independently by a gynecologic oncologist and board-certified radiologist without relevant knowledge of tumor diagnosis to reduce bias in evaluating derived radiomic features. They manually delineated each target lesion along the lesion’s contour and separately extracted calcifications and fats using the CT thresholds described above. Before radiomic feature computation, voxel intensities were resampled using the relative resampling method with a fixed bin number of 128. Forty-eight texture features were extracted, including histogram-based, shape-based, gray-level co-occurrence (GLCM), gray-level run-length, neighborhood gray-level dependence, and gray-level zone length matrixes.

### Reference standard

Histopathological results were used as the reference standard.

### Statistical analysis

The Mann–Whitney U test was used to analyze the relationships between clinical characteristics, CT findings, and pathological findings. Significance was set at *p* < 0.05 (two-sided testing). All statistical analyses were performed using SPSS version 24 (IBM, Armonk, NY, USA).

## Results

### Patients

Table 1 summarizes the clinical information of the 32 included patients. The histopathological subtypes included mature cystic teratoma (*n* = 28), and grades 1 (*n* = 2), 2 (*n* = 1), and 3 immature teratomas (*n* = 1). Moreover, 26 (81.2%) patients were symptomatic with abdominal pain and distension, and 6 (18.8%) were asymptomatic.

**Table 1:** Patient and tumor characteristics

Characteristic	n	%
Total number of patients	32	100
Histology		
mature teratoma	28	87.5
immature teratoma		
grade 1	2	6.3
grade 2	1	3.1
grade 3	1	3.1
Symptoms		
abdominal pain	21	65.6
abdominal distension	5	15.6
none	6	18.8

### Correlation between mature cystic and immature teratomas and clinical parameters

Mature cystic and immature teratomas were correlated with clinical parameters to determine the clinical and pathological impacts. No significant differences in age, height, weight, and BMI were observed between mature cystic and immature teratomas. The mean maximum diameter of immature teratomas (21.5 cm, range: 18.0–25.0) was significantly larger than that of mature cystic teratomas (10.3 cm, 3.0–27.0) (*p* < 0.01). Moreover, the mean diameter of the immature teratomas (15.8 cm, 14.3–17.7) was significantly larger than that of the mature cystic teratomas (8.7 cm, 2.7–18.3) (*p* < 0.01). The mean volume of immature teratomas (3451 cm^3^, 2660–4320) was significantly larger than that of mature cystic teratomas (969.7 cm^3^, 18.8–5049) (*p* < 0.01). No significant differences in symptoms caused by tumors were observed between mature cystic and immature teratomas. Regarding tumor markers, the mean value of AFP for immature teratomas (101.7 ng/mL, 3.8–185.2) was significantly higher than that of mature cystic teratomas (2.1 ng/mL, 0.6–5.7) (*p* < 0.01). Additionally, the mean value of CA125 for immature teratomas (289.4 U/mL, 30.6–885.0) was significantly higher than that of mature cystic teratomas (36.2 U/mL, 8.8–173.9) (*p* < 0.01). However, no significant difference in CA19-9 was observed between mature cystic and immature teratomas (Table 2).

**Table 2:** Differential diagnosis between mature and immature teratoma according to various clinical factors

	mature	immature	*p*-value
Number of patients	28	4	
Age (years)			
means (range)	14.5 (6–19)	14.0 (11–18)	*p* > 0.05
Height (cm)			
means (range)	155.1 (117–168)	152.0 (142–158)	*p* > 0.05
Weight (kg)			
means (range)	49.0 (21–63)	46.0 (34–53)	*p* > 0.05
BMI			
means (range)	20.1 (15.3–25.2)	19.8 (16.9–21.2)	*p* > 0.05
Maximum diameter (cm)			
means (range)	10.3 (3.0–27.0)	21.5 (18.0–25.0)	*p* < 0.01
Mean diameter (cm)			
means (range)	8.7 (2.7–18.3)	15.8 (14.3–17.7)	*p* < 0.01
Volume (cm^3^)			
means (range)	969.7 (18.8–5049)	3451 (2660–4320)	*p* < 0.01
Symptoms			
symptomatic (%)	22 (78.6)	4 (100)	*p* > 0.05
AFP (ng/mL)			
means (range)	2.1 (0.6–5.7)	101.7 (3.8–185.2)	*p* < 0.01
CA125 (U/mL)			
means (range)	36.2 (8.8–173.9)	289.4 (30.6–885.0)	*p* < 0.05
CA19-9 (U/mL)			
means (range)	135.6 (3.6–1350)	406.3 (24.8–1438)	*p* > 0.05

### Correlation between mature cystic and immature teratomas and CT features

Mature cystic and immature teratomas were correlated with the imaging features to determine the imaging and pathological impact. In patients with 10 mature cystic and 4 immature teratomas who underwent abdominal and pelvic CT with a 3 mm slice thickness, the maximum diameter and number of calcifications and fats were manually measured, counted, and evaluated. The mean maximum diameter of calcifications of immature teratomas (48.0 mm, 21.0–63.0) was significantly larger than that of mature cystic teratomas (14.8 mm, 5.0–28.0) (*p* < 0.01). Moreover, the mean number of calcifications of immature teratomas (104.5, 30.0–250.0) was significantly larger than that of mature cystic teratomas (6.8, 1.0–38.0) (*p* < 0.01). Furthermore, the mean number of fats of immature teratomas (63.5, 25.0–100.0) was significantly larger than that of mature cystic teratomas (6.2, 1.0–32.0) (*p* < 0.01). However, no significant difference in the maximum diameter of fats was observed between mature cystic and immature teratomas (Table 3 and Supplementary table 1).

**Table 3:** Differential diagnosis between mature and immature teratoma according to maximum diameter or numbers of calcifications or fat in tumors

	mature	immature	*p*-value
Number of patients	10	4	
Maximum diameter of calcifications (mm)			
means (range)	14.8 (5.0–28.0)	48.0 (21.0–63.0)	*p* < 0.01
Number of calcifications			
means (range)	6.8 (1.0–38.0)	104.5 (30.0–250.0)	*p* < 0.01
Maximum diameter of fats (mm)			
means (range)	40.2 (4.0–74.0)	21.3 (15.0–27.0)	*p* > 0.05
Number of fats			
means (range)	6.2 (1.0–32.0)	63.5 (25.0–100.0)	*p* < 0.01

### Correlation between mature cystic and immature teratomas and CT-based radiomic features

CT features of calcifications of mature cystic and immature teratomas showed that among the six features derived from the GLCM, mature cystic teratoma showed higher homogeneity and energy than immature teratomas (*p* < 0.05). However, immature teratomas showed higher correlation, entropy, and dissimilarity than mature cystic teratomas (*p* < 0.05). Moreover, no significant difference in contrast was observed between mature cystic and immature teratomas. Among the three features derived from the neighborhood grey-level dependence matrix, mature cystic teratomas showed higher coarseness than immature teratomas (*p* < 0.05); however, immature teratomas showed higher contrast and busyness than mature cystic teratomas (*p* < 0.05) (Table 4 and Supplementary tables 2 and 4). Regarding CT features of fats of mature cystic and immature teratomas, no significant differences in six and three features derived from GLCM and neighborhood gray-level dependence matrix were observed between mature cystic immature teratomas (Table 5 and supplementary tables 3 and 5).

**Table 4:** Comparison of CT features of calcifications between mature and immature teratoma

Parent matrix	Feature	mature		immature		*p*-value
		mean	SEM	mean	SEM	
Gray-level co-occurrence matrix (GLCM)	Homogeneity	0.9971	0.00138	0.9898	0.00157	*p* < 0.05
	Energy	0.9929	0.00326	0.9721	0.00434	*p* < 0.05
	Contrast	0.9679	0.66601	0.7807	0.27656	*p* > 0.05
	Correlation	0.5085	0.04363	0.6386	0.00606	*p* < 0.05
	Entropy	0.0596	0.02675	0.2072	0.03	*p* < 0.05
	Dissimilarity	0.0414	0.024	0.0742	0.01496	*p* < 0.05
Neighborhood grey-level dependence matrix (NGLDM)	Coarseness	0.0013	0.00047	0.0001	0.00003	*p* < 0.05
	Contrast	0.0001	0.00005	0.0001	0.00002	*p* < 0.05
	Busyness	57.8466	31.36417	407.1174	142.80582	*p* < 0.05

**Table 5:** Comparison of CT features of fats between mature and immature teratoma

Parent matrix	Feature	mature		immature		*p*-value
		mean	SEM	mean	SEM	
Gray-level co-occurrence matrix (GLCM)	Homogeneity	0.8801	0.04603	0.9797	0.00859	*p* > 0.05
	Energy	0.7669	0.07594	0.9541	0.01823	*p* > 0.05
	Contrast	98.9387	40.36167	15.1771	8.921	*p* > 0.05
	Correlation	0.6279	0.06626	0.4795	0.05858	*p* > 0.05
	Entropy	1.9599	0.71651	0.3611	0.14955	*p* > 0.05
	Dissimilarity	2.5729	1.03476	0.4291	0.23335	*p* > 0.05
Neighborhood grey-level dependence matrix (NGLDM)	Coarseness	0.0001	0.00003	0	0.00001	*p* > 0.05
	Contrast	0.1382	0.08001	0.0021	0.00184	*p* > 0.05
	Busyness	6.7305	3.8123	8.8092	5.42011	*p* > 0.05

### Representative cases

**Fig. 1:**
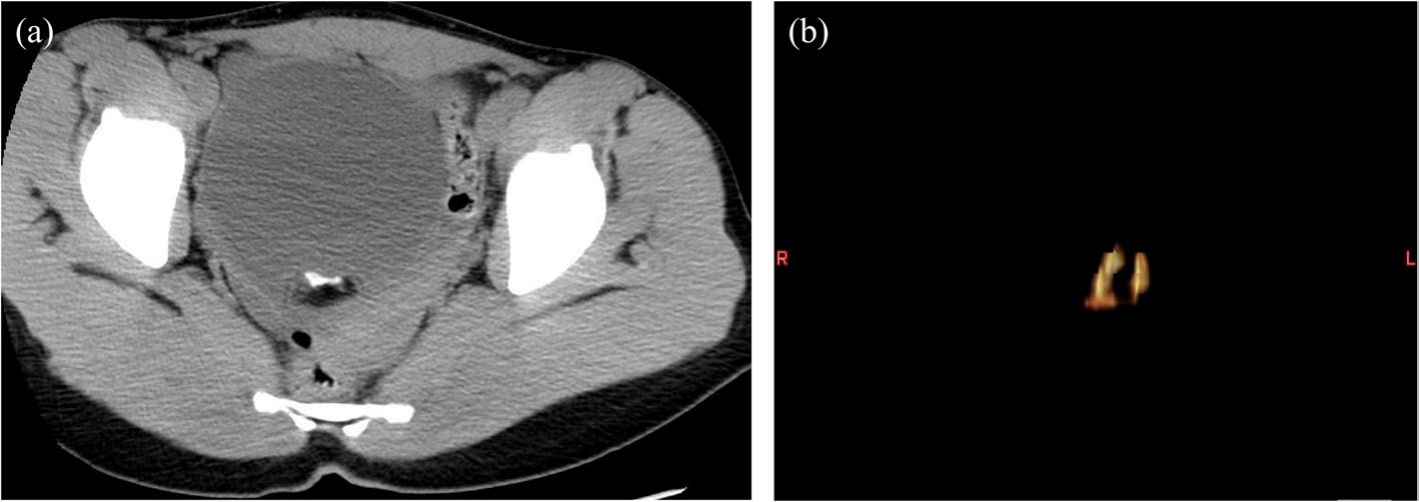
A 17-year-old female with mature cystic teratoma. **a** CT image shows the calcification in the tumor or cyst wall filled with sebaceous material. **b** A three-dimensional image shows a few small calcifications

**Fig. 2:**
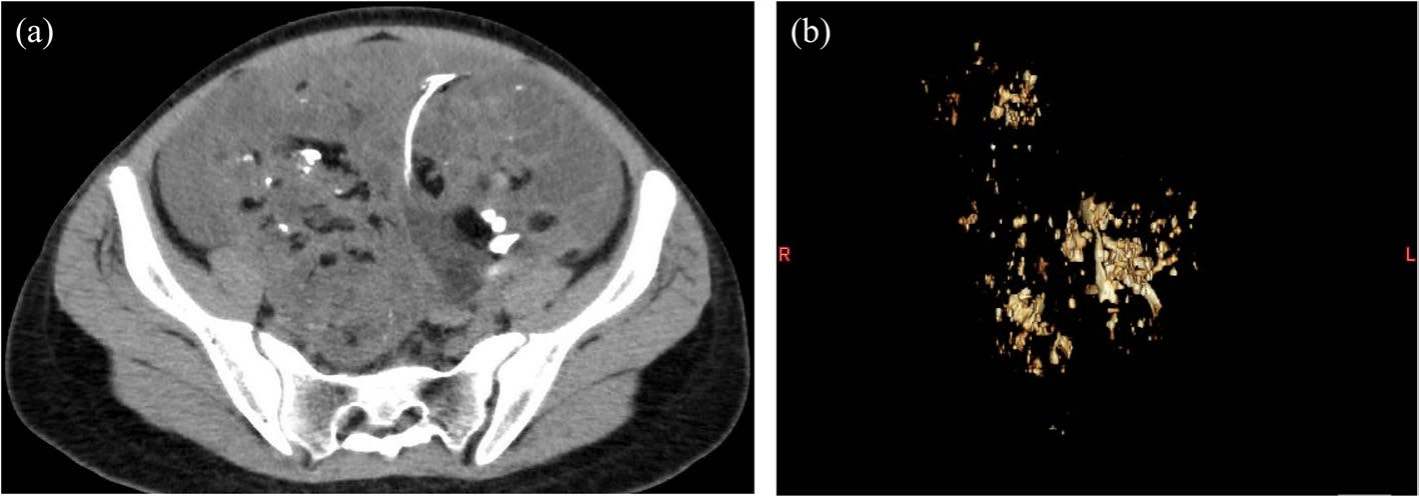
A 14-year-old female with immature teratoma. **a** CT image shows the scattered calcifications and small fatty areas in the tumor. **b** A three-dimensional image shows the scattered calcifications of various sizes

Figures 1 and 2 show two representative cases. Figure 1a and b show CT and the three-dimensional image, respectively, with the distribution of calcifications in a mature cystic teratoma. Moreover, Fig. 2a and b show CT and the three-dimensional image, respectively, with the distribution of calcifications in an immature teratoma. Immature teratomas showed more calcifications than mature cystic teratomas.

## Discussion

To our knowledge, this is the first study to investigate the application of CT-based textural analysis in discriminating mature cystic teratomas from immature teratomas. Subjective CT assessment showed that immature teratomas had more and larger calcifications than mature teratomas, supported by the CT-based textural analysis. Furthermore, the CT-based textural features of mature and immature teratoma calcifications showed that mature cystic teratomas had higher homogeneity and energy than immature teratomas. However, immature teratomas showed higher correlation, entropy, and dissimilarity than mature cystic teratomas. These findings suggest that calcification distribution in tumors can be a useful diagnostic biomarker to distinguish between mature and immature teratomas, facilitating the optimal therapeutic selection in patients < 20 years.

The most frequent type of germ cell tumor is teratoma, mostly the benign mature type. However, in patients < 20 years, > 40% of germ cell tumors are malignant [1] and require at least adnexectomy even in the early stage of the disease [3]. Therefore, to preserve fertility, accurate and prompt preoperative differential diagnostic strategies for mature and malignant immature teratomas are necessary, particularly in emergencies where patients show symptoms due to torsion or rupture.

We first evaluated the relationship between clinical features and pathological findings. We observed no significant differences in clinical parameters, including age, height, weight, BMI, and symptoms, such as abdominal pain or distension. Regarding tumor markers, AFP and CA125 levels were significantly higher in immature teratomas than in mature teratomas. Additionally, CA19-9 of immature teratomas was higher than that of mature teratomas; however, the difference was not significant because CA19-9 is sometimes high, even in benign mature teratomas. These results are consistent with those of previous studies. Therefore, AFP and CA125 are useful biomarkers for differentiating mature from immature teratomas [4]. However, many general hospitals cannot measure these tumor markers during off-hours. Therefore, another diagnostic tool is needed for early and prompt diagnosis, particularly in emergencies.

CT is often used to diagnose the cause of sudden abdominal pain. The size and appearance of the tumors are evaluated in the differential diagnosis between mature and immature teratomas using CT. Mature teratomas reportedly have an average tumor diameter of 7 cm; however, immature teratomas are larger, with a diameter of 12–25 cm [5]. Similarly, in our study, the size of immature teratoma (21.5 cm, 18.0–25.0) was significantly larger than that of mature teratoma (10.3 cm, 3.0–27.0). Moreover, in our study, the mature teratomas were larger than previously reported ones. As asymptomatic patients aged < 20 years do not often visit the hospital for medical examinations, the tumor size might become large, leading to difficulty in differential diagnosis using only tumor sizes. These findings are consistent with the high rate of symptomatic patients with mature and immature teratomas. The presence of a prominent solid component with cystic elements, such as epithelial ovarian cancer or microcystic appearance of solid components on CT or MRI, is caused by respiratory-type epithelial tissues [8]. However, in our study, immature teratomas did not have epithelial ovarian cancer or microcystic appearances; hence, the need for another biomarker to differentiate between mature and immature teratomas.

Calcification or fat distribution in tumors is reportedly different; immature teratomas show scattered calcifications and small fatty areas in the tumors, and mature teratomas show only a few calcifications in the cyst wall [6–8]. However, reports of quantifying these distributions for differential diagnosis are lacking. First, we manually counted the number of calcifications and fats in the tumor and measured their maximum diameter. Consistent with previous findings, the number of calcifications and fats in immature teratomas was significantly larger than that in mature teratomas. Moreover, the maximum diameter of calcifications of immature teratomas was significantly larger than that of mature teratomas because immature teratomas showed scattered calcifications of variable size. Although these findings could provide a precise diagnosis to differentiate between immature and mature teratomas, the objective quantification of calcification and fat distributions between these tumors has not been reported.

Texture analysis is a method of assessing the local and regional heterogeneities of distribution with textural feature measurements, using various mathematical methods that describe the relationships between the gray-level intensity of pixels or voxels and their position within an image. Texture analysis has been used to differentiate between benign and malignant lesions in different organs, including epithelial ovarian cancer [9, 13, 14]. In this study, we applied CT-based texture analysis of calcification or fat distribution to distinguish between mature and immature teratomas. In particular, we focused on the homogeneity or dissimilarity derived from the GLCM to demonstrate the difference of the distribution of calcification or fat objectively. Mature cystic teratomas showed higher homogeneity and energy than immature teratomas. However, immature teratomas showed higher correlation, entropy, and dissimilarity than mature cystic teratomas among features derived from the GLCM (*p* < 0.05), suggesting that immature teratomas had a more scattered and heterogeneous distribution of calcifications compared with mature teratomas, which had more homogeneous distribution. These findings were consistent with our subjective results obtained by manually counting the number of calcifications. No significant differences were observed in the CT features of fats derived from GLCM. The difference between the subjective method of manually counting the number of fats and the objective method of texture analysis might be due to the difficulty in counting the fat manually because the CT density of fats was closer to that of the surrounding soft tissue compared with that of calcifications.

This study has some limitations. First, this study was retrospective, and not all patients underwent CT examinations at our institution. However, our readers reevaluated the images from other hospitals and were blinded to the initial imaging findings. Second, because of the disease rarity, the sample size was small, particularly for immature teratomas. Therefore, further prospective and multicenter studies are needed. Third, textural analysis cannot be used in daily practice. Therefore, we manually counted the number of calcifications and fats in the tumor and measured their maximum diameter and demonstrated that these results were consistent with the results of texture analysis..

In conclusion, calcification distribution on CT can be a potential diagnostic biomarker to discriminate mature teratomas from immature teratomas. This was objectively demonstrated by CT-based textural analysis, enabling optimal therapeutic selection for patients aged < 20 years.

### Supplementary Information

**Additional file 1: Table 1.** Differential diagnosis between mature and immature teratoma according to maximum diameter or numbers of calcifications or fat in tumors. **Table 2.** Comparison of CT features of calcifications between mature and immature teratoma. **Table 3.** Comparison of CT features of fats between mature and immature teratoma. **Table 4.** Comparison of other CT features of calcifications between mature and immature teratoma. **Table 5.** Comparison of other CT features of fats between mature and immature teratoma.

## Data Availability

The data supporting the findings of this study are available within the article.

## References

[1] Brammer HM, Buck JL, Hayes WS, Sheth S, Tavassoli FA (1990). From the archives of the AFIP Malignant germ cell tumors of the ovary: radiologic-pathologic correlation. Radiographics..

[2] Outwater EK, Siegelman ES, Hunt JL (2001). Ovarian teratomas: tumor types and imaging characteristics. Radiographics.

[3] Nasioudis D, Mastroyannis SA, Latif NA, Ko EM (2020). Trends in the surgical management of malignant ovarian germcell tumors. Gynecol Oncol.

[4] Kawai M, Kano T, Kikkawa F, Morikawa Y, Oguchi H, Nakashima N (1992). Seven tumor markers in benign and malignant germ cell tumors of the ovary. Gynecol Oncol.

[5] Park SB, Kim JK, Kim KR, Cho KS (2008). Imaging findings of complications and unusual manifestations of ovarian teratomas. Radiographics.

[6] Saba L, Guerriero S, Sulcis R, Virgilio B, Melis G, Mallarini G (2009). Mature and immature ovarian teratomas: CT, US and MR imaging characteristics. Eur J Radiol.

[7] Choudhary S, Fasih N, Mc Innes M, Marginean C (2009). Imaging of ovarian teratomas: appearances and complications. J Med Imaging Radiat Oncol.

[8] Yamaoka T, Togashi K, Koyama T, Fujiwara T, Higuchi T, Iwasa Y (2003). Immature teratoma of the ovary: correlation of MR imaging and pathologic findings. Eur Radiol.

[9] Bianconi F, Palumbo I, Fravolini ML, Rondini M, Minestrini M, Pascoletti G, et al. Form Factors as Potential Imaging Biomarkers to Differentiate Benign vs. Malignant Lung Lesions on CT Scans. Sensors (Basel). 2022;22(13):5044. 10.3390/s22135044 PMC9269784 35808538

[10] Stamoulou E, Spanakis C, Manikis GC, Karanasiou G, Grigoriadis G, Foukakis T, et al. Harmonization Strategies in Multicenter MRI-Based Radiomics. J Imaging. 2022;8(11)303. 10.3390/jimaging8110303 PMC9695920 36354876

[11] Pineiro-Fiel M, Moscoso A, Pubul V, Ruibal A, Silva-Rodriguez J, Aguiar P. A Systematic Review of PET Textural Analysis and Radiomics in Cancer. Diagnostics (Basel). 2021;11(2)380. 10.3390/diagnostics11020380 PMC7926413 33672285

[12] Lubner MG, Smith AD, Sandrasegaran K, Sahani DV, Pickhardt PJ (2017). CT Texture Analysis: Definitions, Applications, Biologic Correlates, and Challenges. Radiographics.

[13] Tsujikawa T, Yamamoto M, Shono K, Yamada S, Tsuyoshi H, Kiyono Y (2017). Assessment of intratumor heterogeneity in mesenchymal uterine tumor by an (18)F-FDG PET/CT texture analysis. Ann Nucl Med.

[14] Nougaret S, McCague C, Tibermacine H, Vargas HA, Rizzo S, Sala E (2021). Radiomics and radiogenomics in ovarian cancer: a literature review. Abdom Radiol (NY).

[15] Agatston AS, Janowitz WR, Hildner FJ, Zusmer NR, Viamonte M, Detrano R (1990). Quantification of coronary artery calcium using ultrafast computed tomography. J Am Coll Cardiol.

[16] Guinet C, Ghossain MA, Buy JN, Malbec L, Hugol D, Truc JB (1995). Mature cystic teratomas of the ovary: CT and MR findings. Eur J Radiol.

[17] Nioche C, Orlhac F, Boughdad S, Reuze S, Goya-Outi J, Robert C (2018). LIFEx: A Freeware for Radiomic Feature Calculation in Multimodality Imaging to Accelerate Advances in the Characterization of Tumor Heterogeneity. Cancer Res.

